# Supplementary figures and images for: Surface anticoagulation of mechanical heart valves using electrically induced biomimetic glycocalyx: An in-vitro study to assess hemocompatibility and optimal voltage
Source: PLoS One. 2026 May 6;21(5):e0336760. doi: 10.1371/journal.pone.0336760 (PMC13148773; doi:10.1371/journal.pone.0336760)

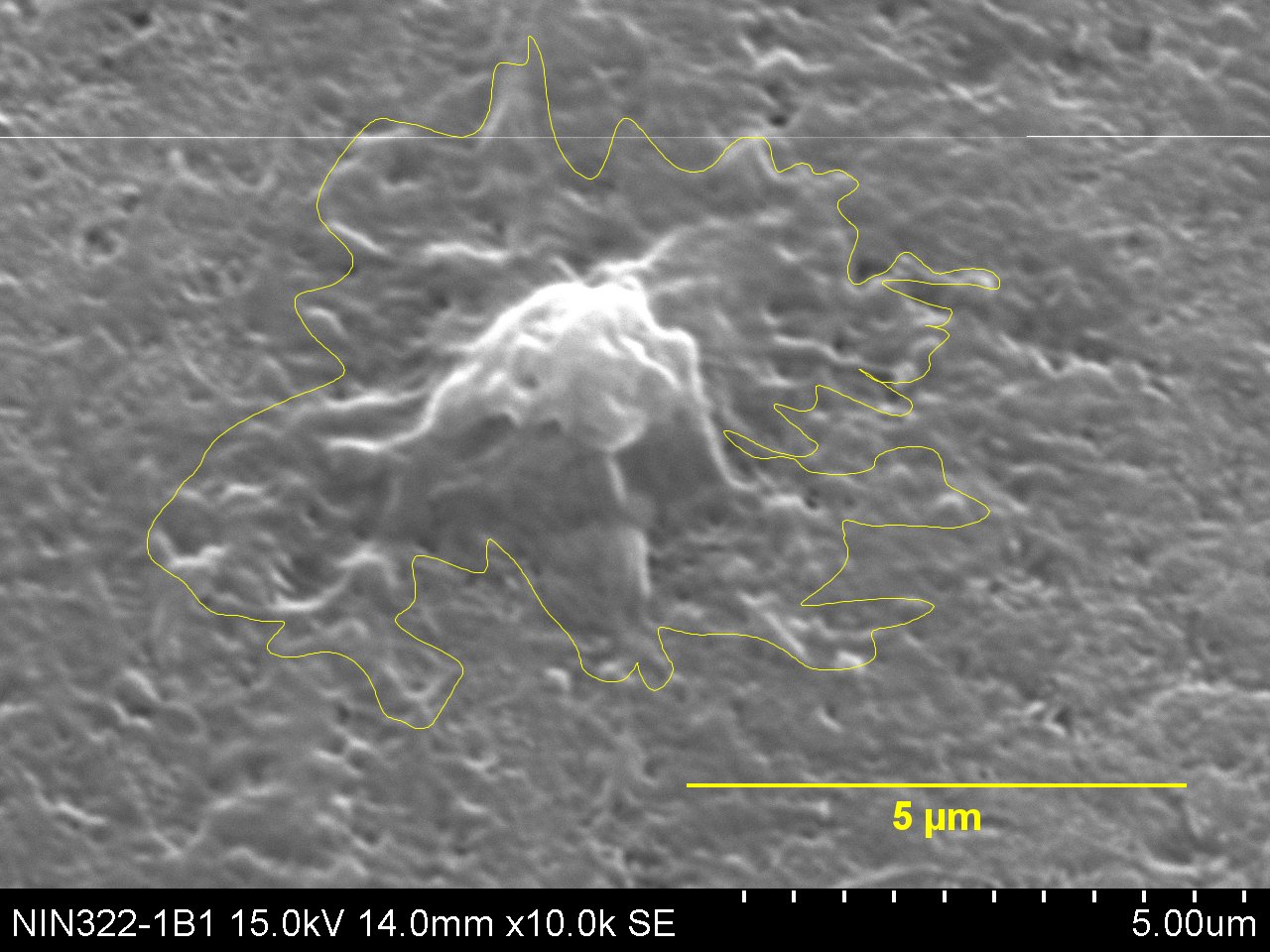

Supplement: S1 Fig — Total area = 112.749 μm2, platelet area = 31.212 μm2 (single platelet in dendritic spreading stage of platelet activation), RBC area = 0, Acellular deposit area = 0, Total area occupied by masses (cells and deposits)=31.212 μm2 (27.68%), Mass free area = 81.537 μm2 (72.32%), Magnification = 10000x, working distance = 14 mm. The scale bar is shown in the image for reference. (TIFF) [file pone.0336760.s001.tiff]

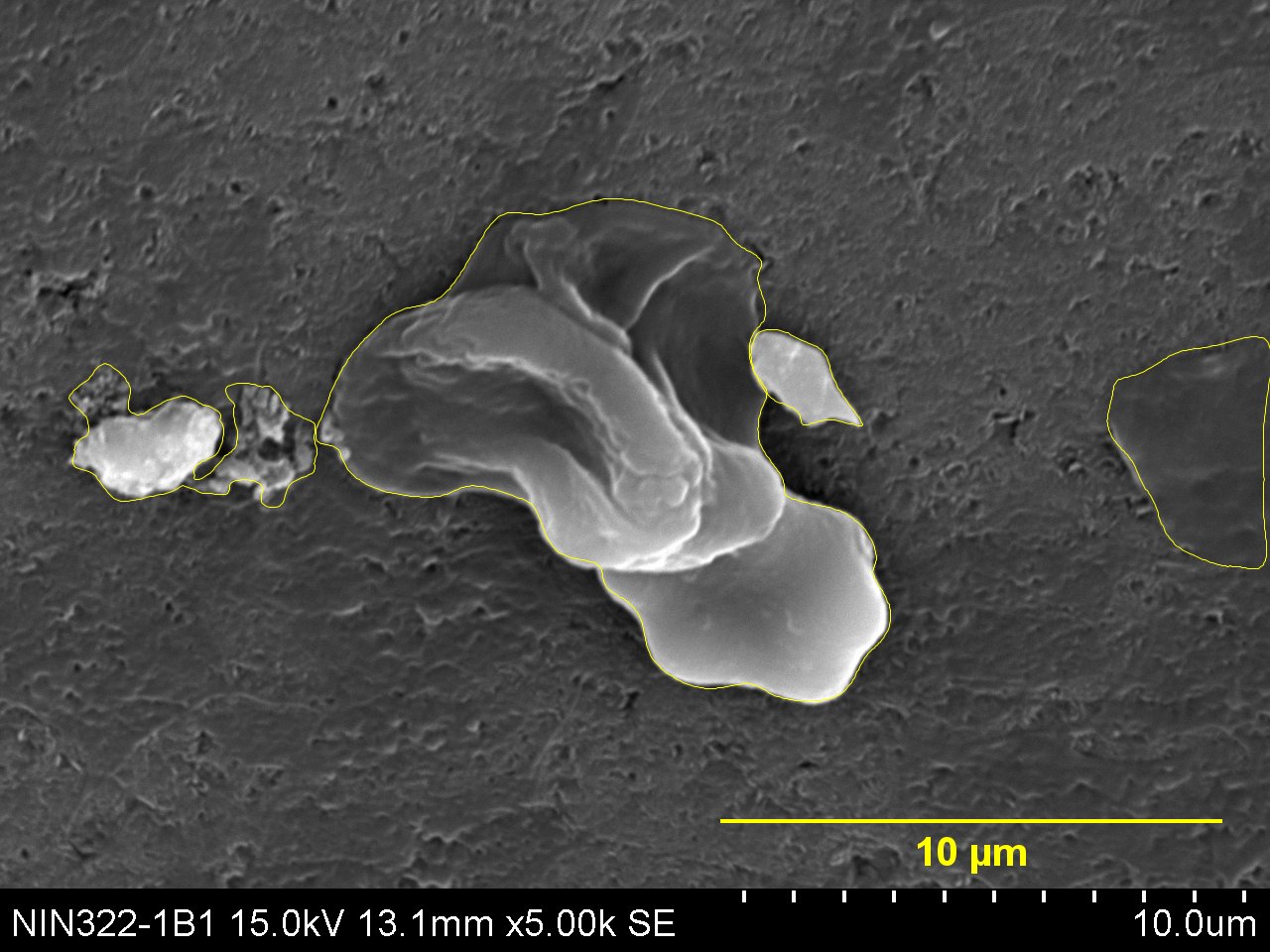

Supplement: S2 Fig — Mass of shrunken RBCs are seen along with amorphous material deposits. Total area = 444.587 μm2, platelet area = 0, RBC area = 62.06 μm2, Acellular deposit area = 21.662 μm2, Total area occupied by masses (cells and deposits)=83.722 μm2 (18.83%), Mass free area = 360.865 μm2 (81.17%), Magnification = 5000x, working distance = 13.1 mm. The scale bar is shown in the image for reference. (TIFF) [file pone.0336760.s002.tiff]

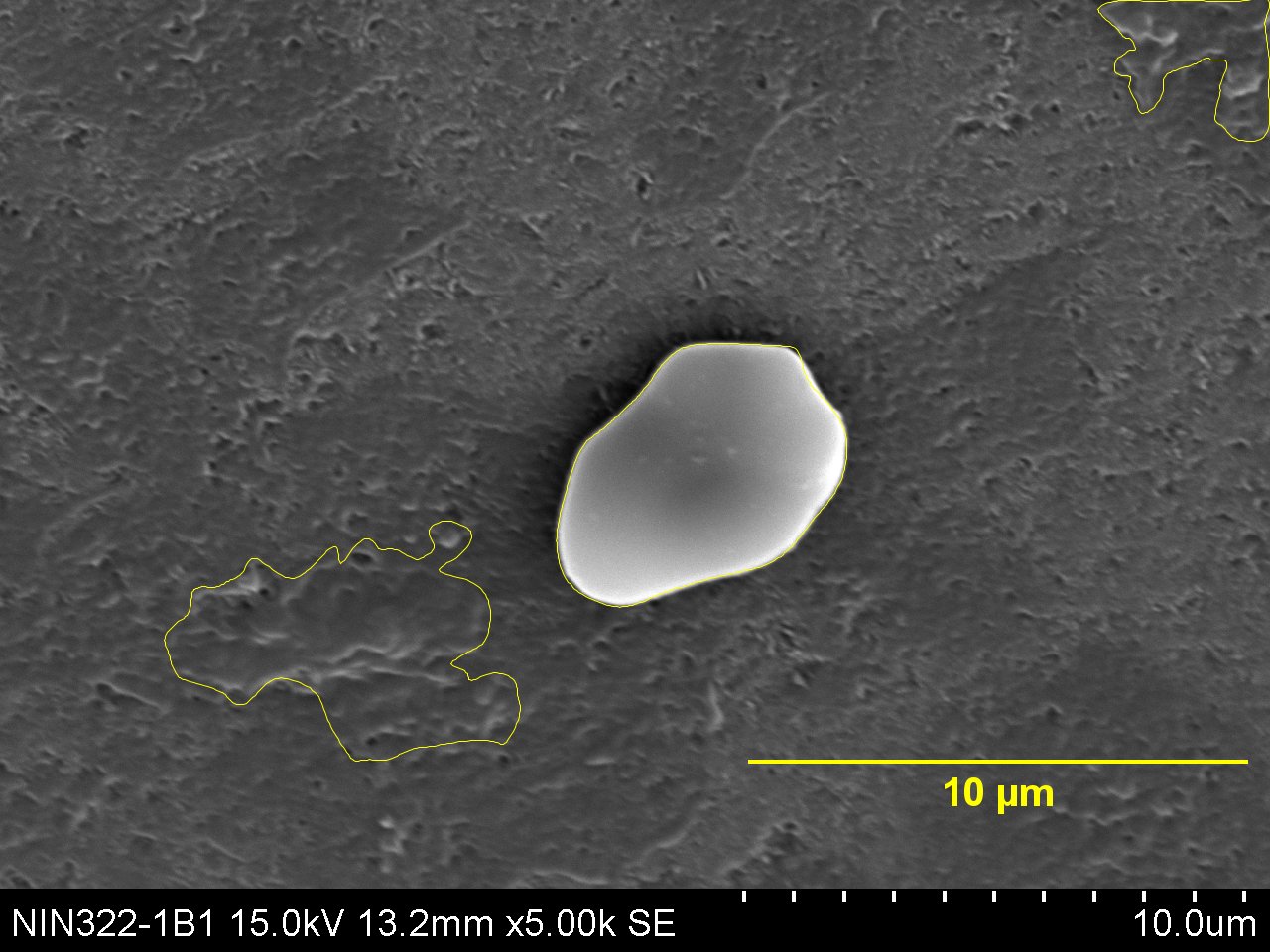

Supplement: S3 Fig — Single deformed RBC is seen along with amorphous material deposits. Total area = 452.001 μm2, platelet area = 0, RBC area = 21.61 μm2, Acellular deposit area = 25.811 μm2, Total area occupied by masses (cells and deposits)=47.421 μm2 (10.49%), Mass free area = 404.58 μm2 (89.51%), Magnification = 5000x, working distance = 13.2 mm. The scale bar is shown in the image for reference. (JPG) [file pone.0336760.s003.jpg]

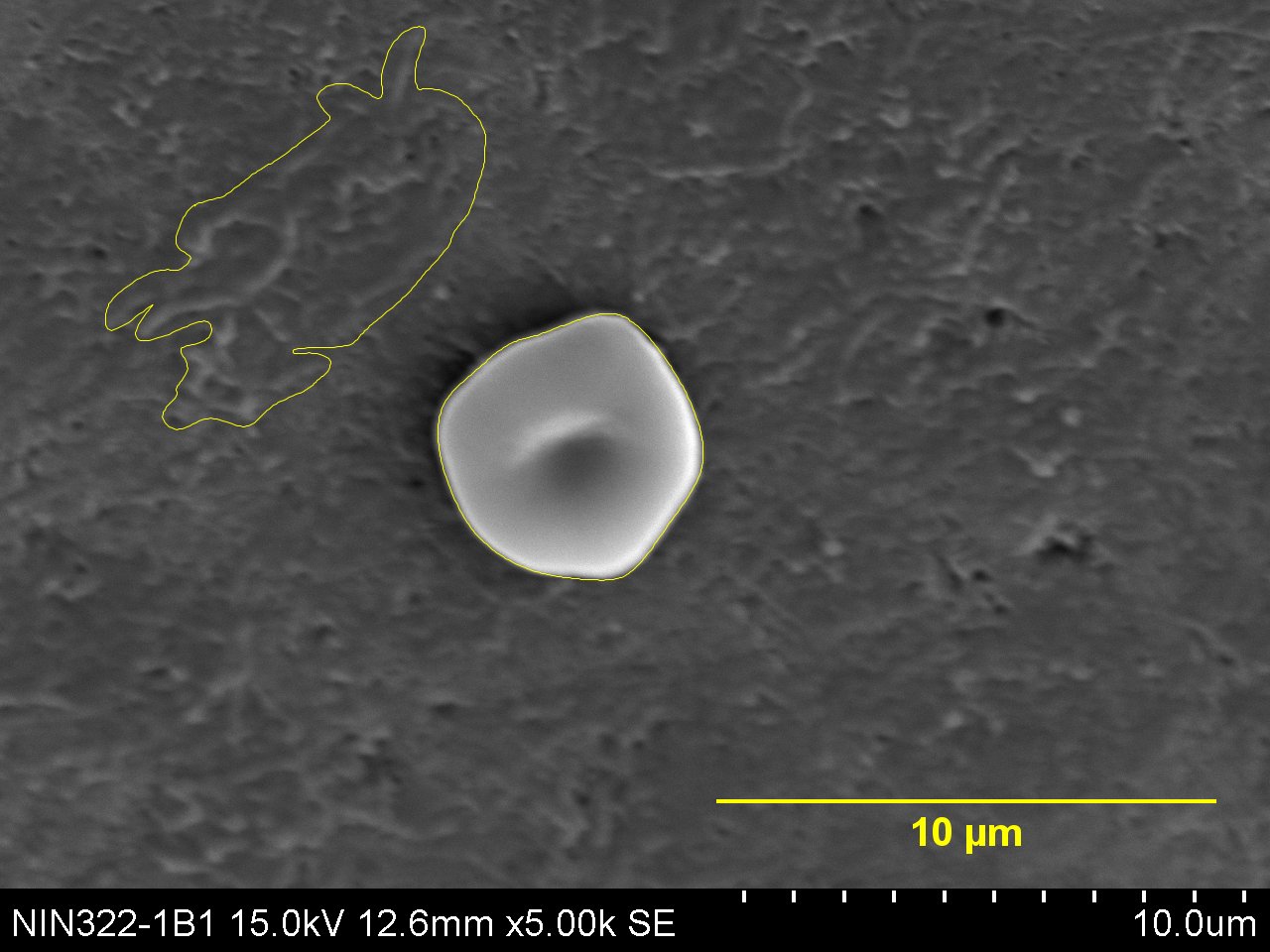

Supplement: S4 Fig — Single RBC is seen along with amorphous material deposits. Total area = 450.614 μm2, platelet area = 0, RBC area = 20.986 μm2, Acellular deposit area = 28.163 μm2, Total area occupied by masses (cells and deposits)=49.149 μm2 (10.91%), Mass free area = 401.465 μm2 (89.09%), Magnification = 5000x, working distance = 12.6 mm. The scale bar is shown in the image for reference. (TIFF) [file pone.0336760.s004.tiff]

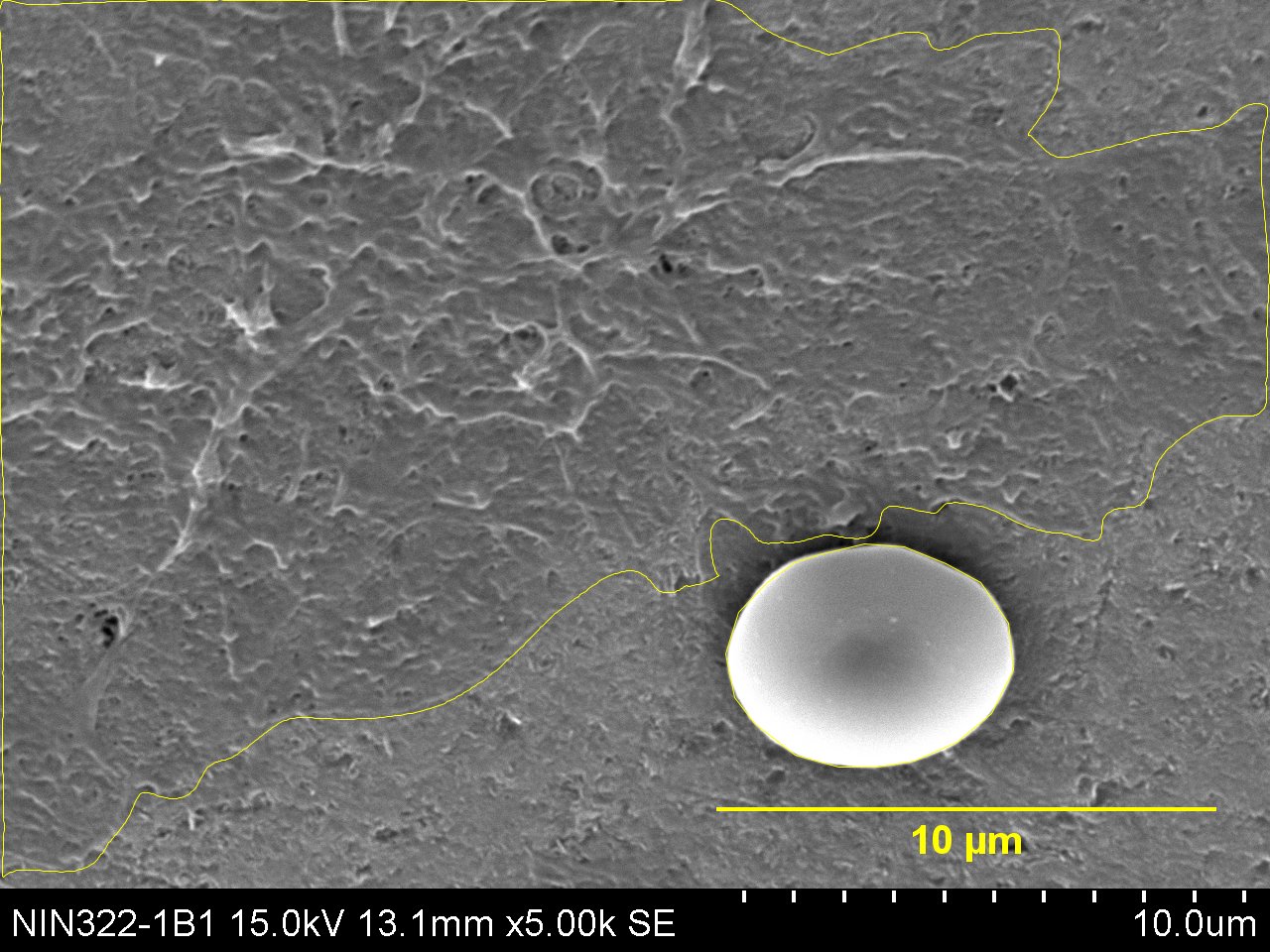

Supplement: S5 Fig — A single RBC, along with large areas of amorphous deposits covering the valve, is seen. Total area = 451.497 μm2, platelet area = 0, RBC area = 19.736 μm2, Acellular deposit area = 298.594 μm2, Total area occupied by masses (cells and deposits)=318.33 μm2 (70.51%), Mass free area = 133.167 μm2 (29.49%), Magnification = 5000x, working distance = 13.1 mm. The scale bar is shown in the image for reference. (TIFF) [file pone.0336760.s005.tiff]

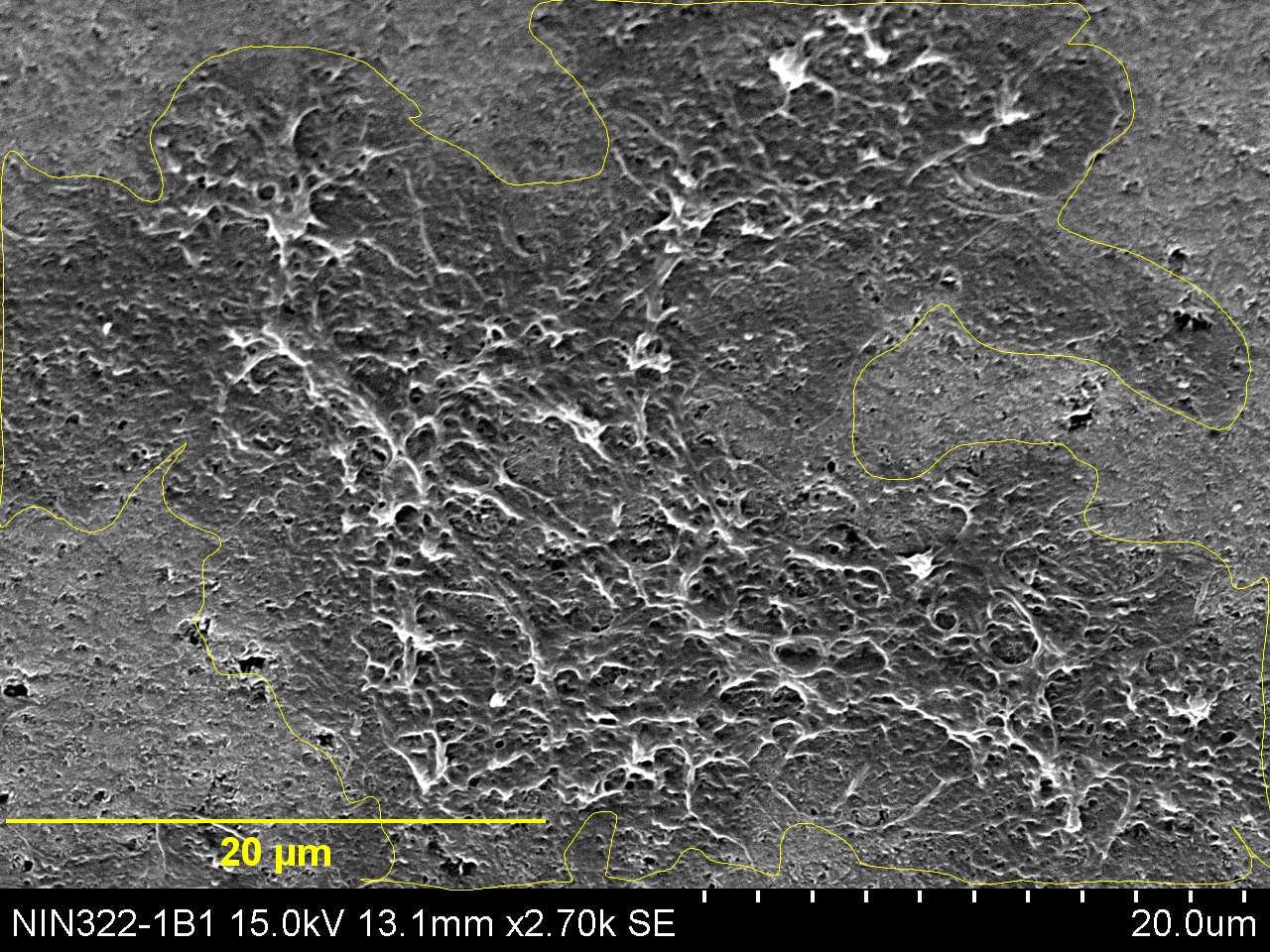

Supplement: S6 Fig — Large areas of amorphous deposits resembling fibrin coagulum covering the valve are seen. Total area = 1553.502 μm2, platelet area = 0, RBC area = 0 μm2, Acellular deposit area = 1138.716 μm2, Total area occupied by masses (cells and deposits)=1138.716 μm2 (73.3%), Mass free area = 414.786 μm2 (26.7%), Magnification = 2700x, working distance = 13.1 mm. The scale bar is shown in the image for reference. (TIFF) [file pone.0336760.s006.tiff]

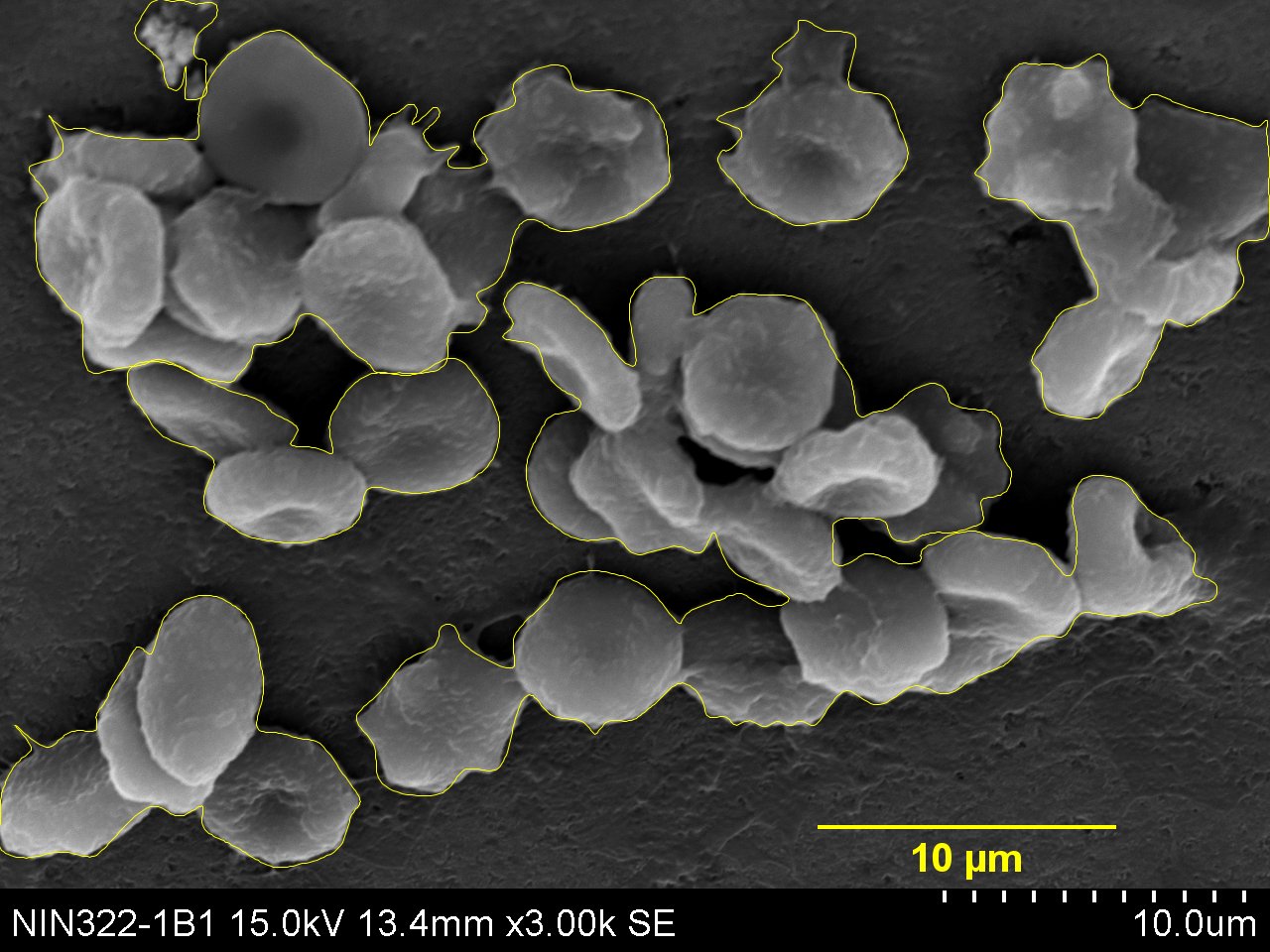

Supplement: S7 Fig — Large numbers of RBCs forming aggregates are seen covering the valve. Total area = 1267.215 μm2, platelet area = 0, RBC area = 570.823 μm2, Acellular deposit area = 5.531 μm2, Total area occupied by masses (cells and deposits)=576.354 μm2 (45.48%), Mass free area = 690.861 μm2 (54.52%), Magnification = 3000x, working distance = 13.4 mm. The scale bar is shown in the image for reference. (TIFF) [file pone.0336760.s007.tiff]

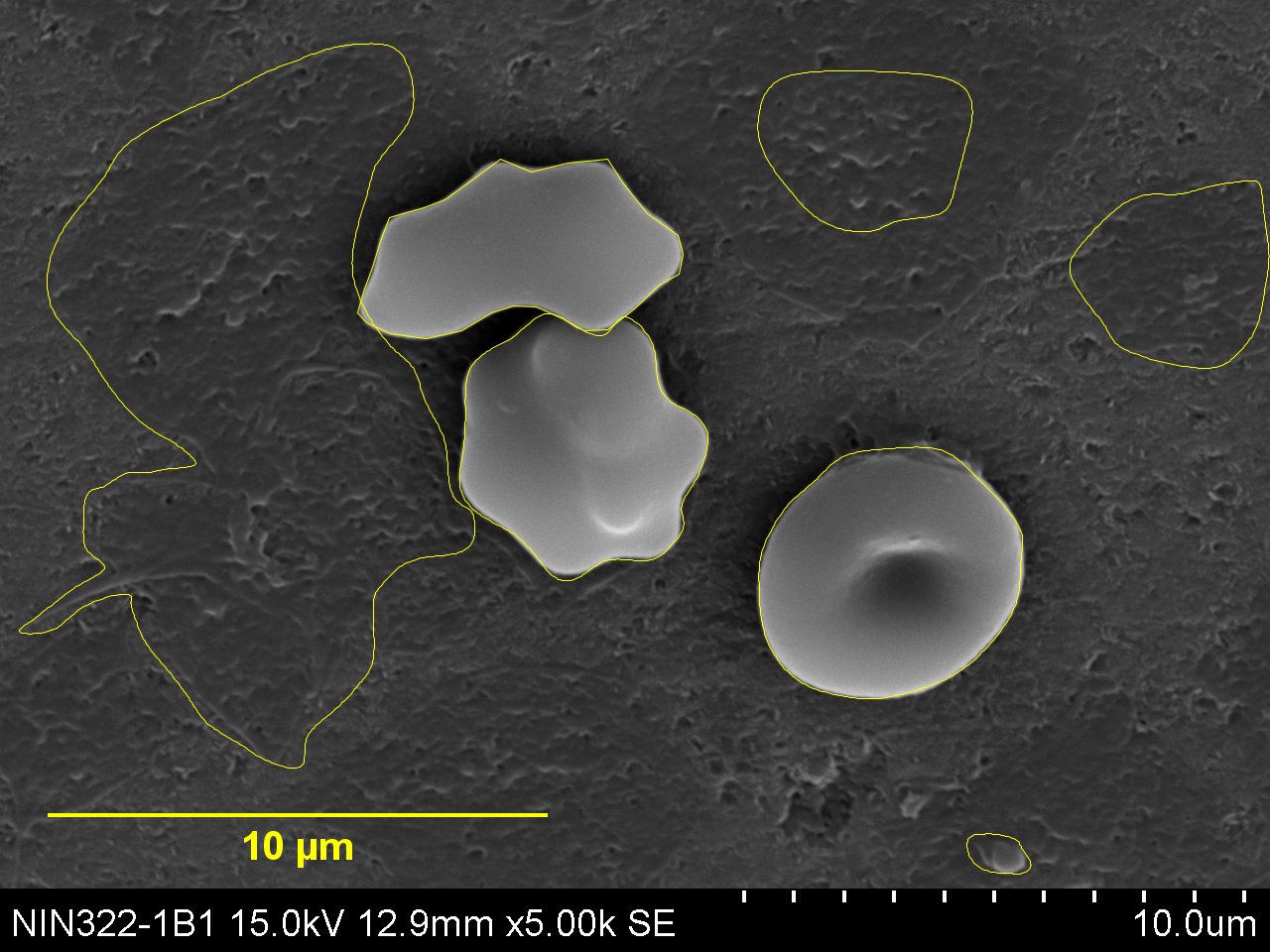

Supplement: S8 Fig — Isolated RBCs and small focal deposits are seen on the valve. Total area = 451.65 μm2, platelet area = 0, RBC area = 56.026 μm2, Acellular deposit area = 98.267 μm2, Total area occupied by masses (cells and deposits)=154.293 μm2 (34.16%), Mass free area = 297.357 μm2 (65.84%), Magnification = 5000x, working distance = 12.9 mm. The scale bar is shown in the image for reference. (TIFF) [file pone.0336760.s008.tiff]

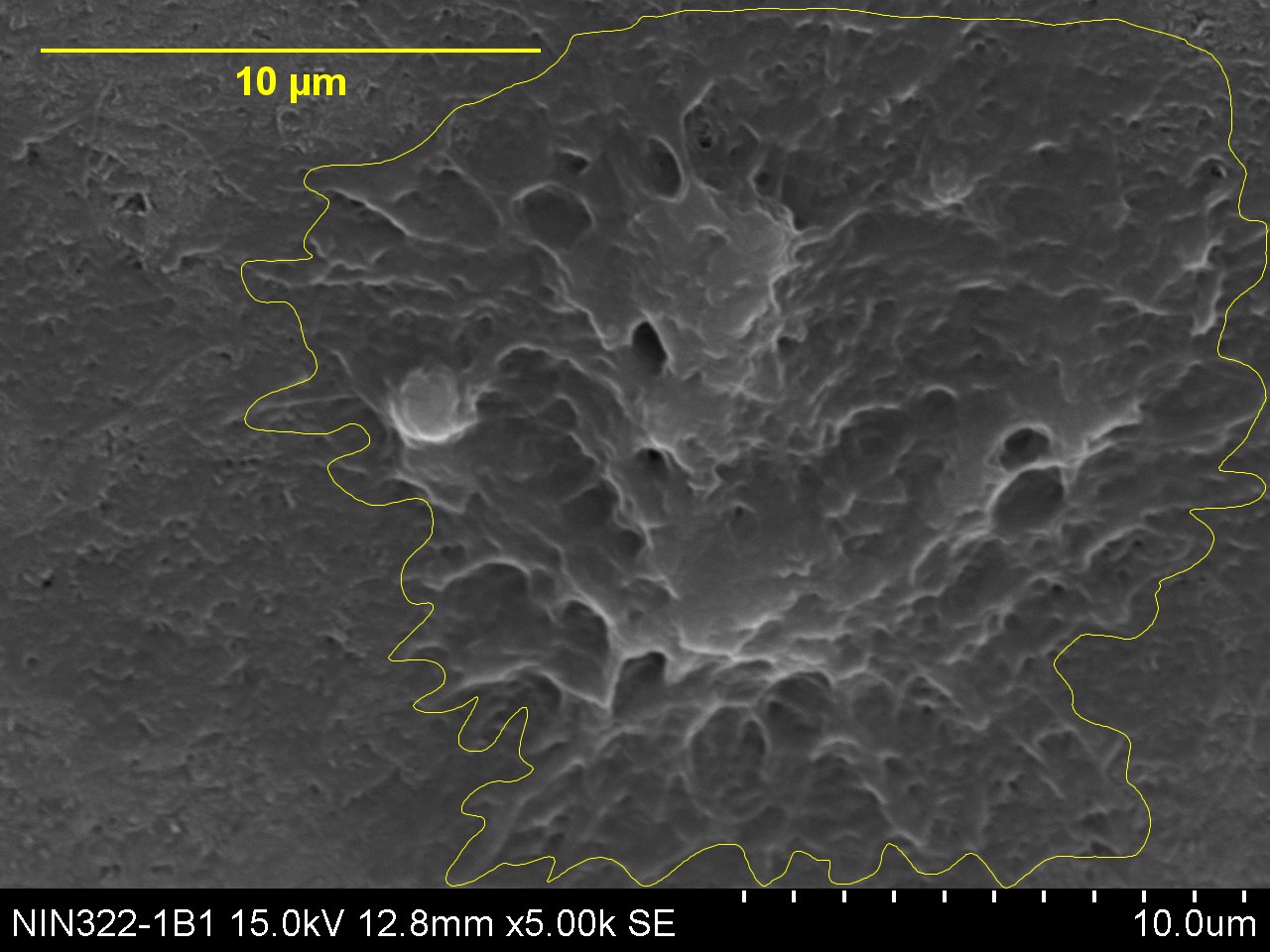

Supplement: S9 Fig — Large amorphous material seen on the valve. Total area = 451.499 μm2, platelet area = 0, RBC area = 0 μm2, Acellular deposit area = 270.202 μm2, Total area occupied by masses (cells and deposits)=270.202 μm2 (59.85%), Mass free area = 181.297 μm2 (40.15%), Magnification = 5000x, working distance = 12.8 mm. The scale bar is shown in the image for reference. (TIFF) [file pone.0336760.s009.tiff]

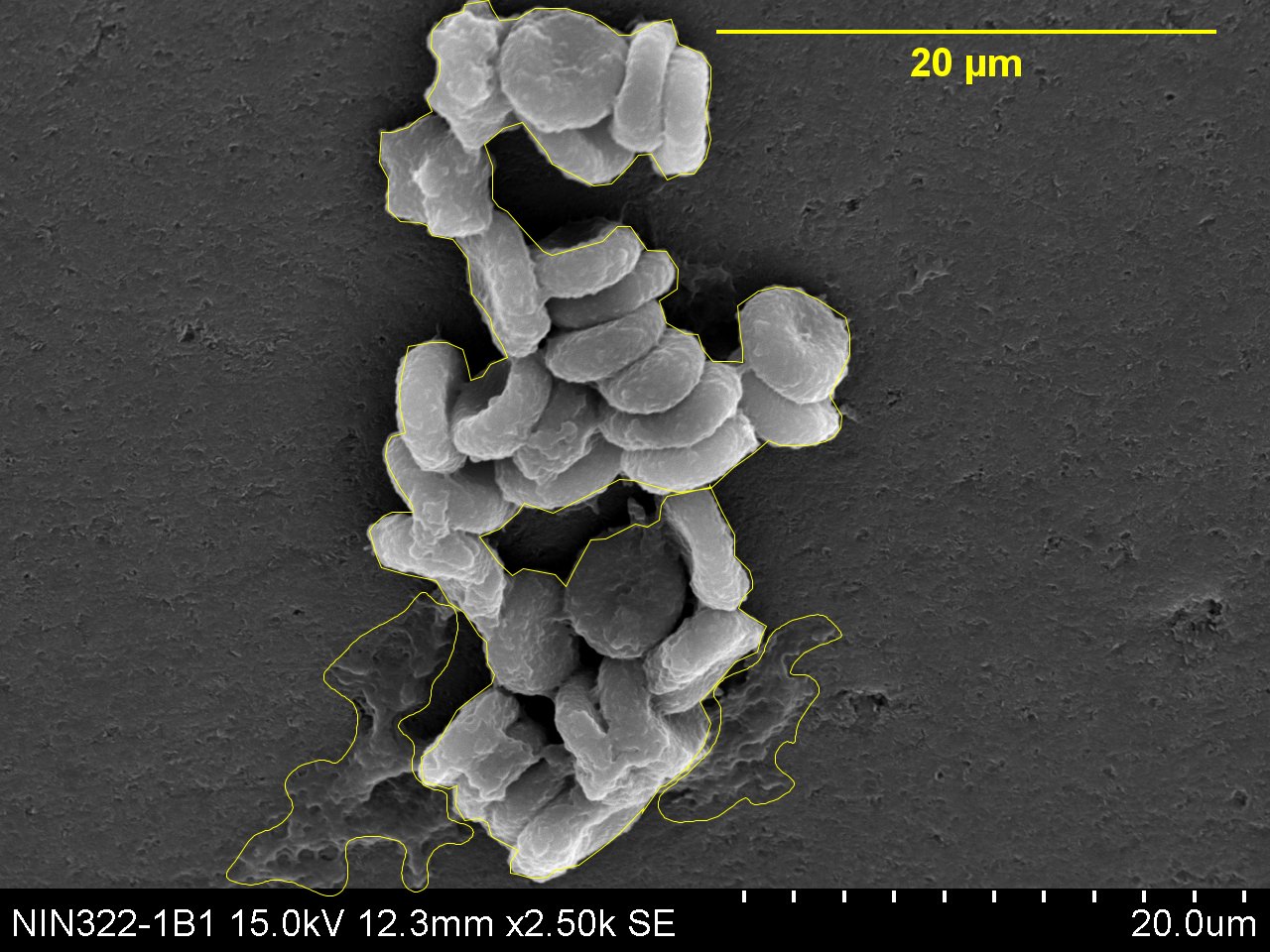

Supplement: S10 Fig — Clusters of RBCs and small focal deposits are seen on the valve. Total area = 1804.586 μm2, platelet area = 0, RBC area = 351.496 μm2, Acellular deposit area = 75.685 μm2, Total area occupied by masses (cells and deposits)=427.181 μm2 (23.67%), Mass free area = 1377.405 μm2 (76.33%), Magnification = 2500x, working distance = 12.3 mm. The scale bar is shown in the image for reference. (TIFF) [file pone.0336760.s010.tiff]

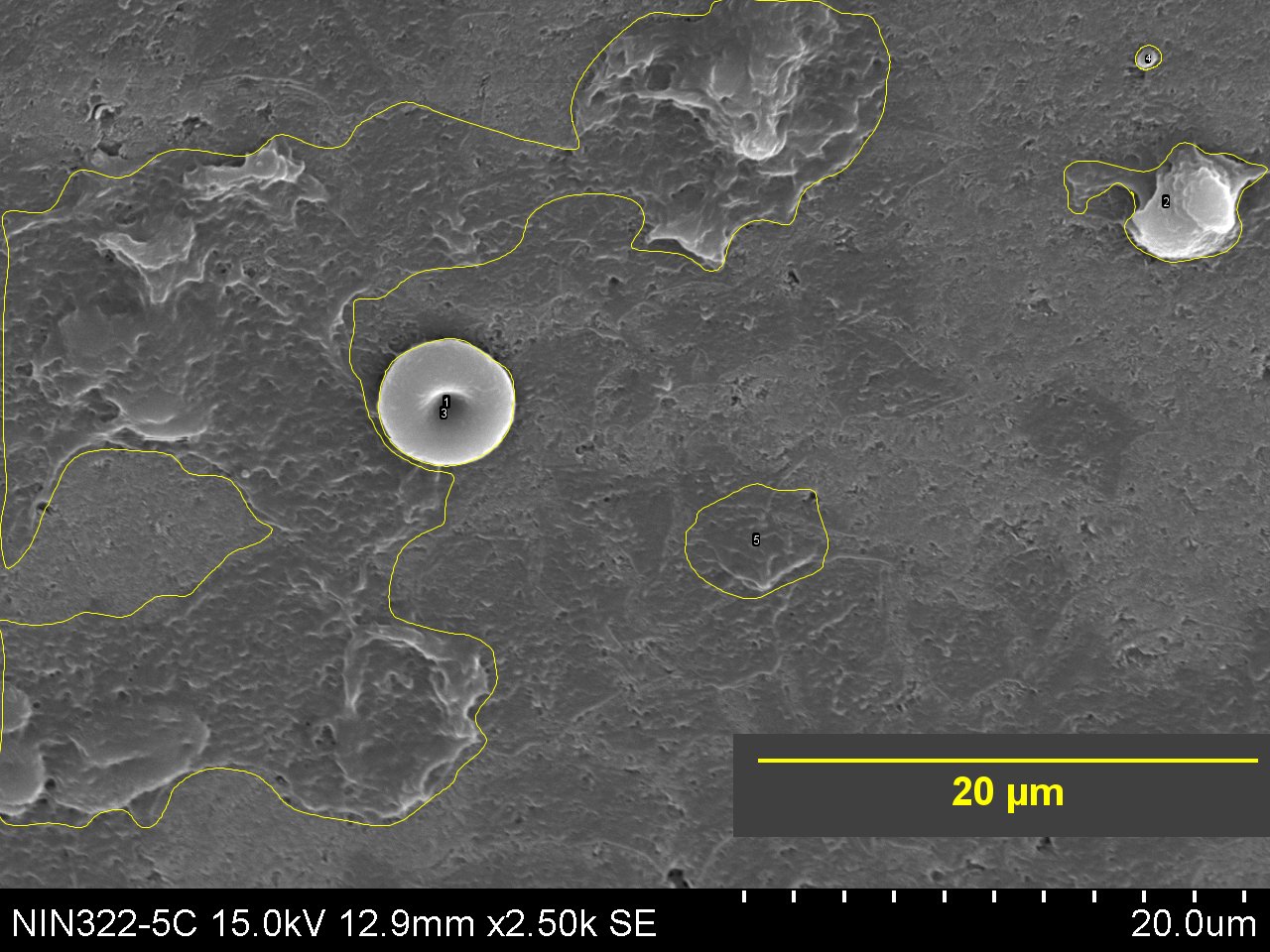

Supplement: S11 Fig — Isolated RBC, smaller platelet aggregate, and amorphous deposits are seen on the valve. Total area = 1800.529 μm2, platelet area = 21.542, RBC area = 21.64 μm2, Acellular deposit area = 529.43 μm2, Total area occupied by masses (cells and deposits)=573.393 μm2 (31.85%), Mass free area = 1227.136 μm2 (68.15%), Magnification = 2500x, working distance = 12.9 mm. The scale bar is shown in the image for reference. (TIFF) [file pone.0336760.s011.tiff]

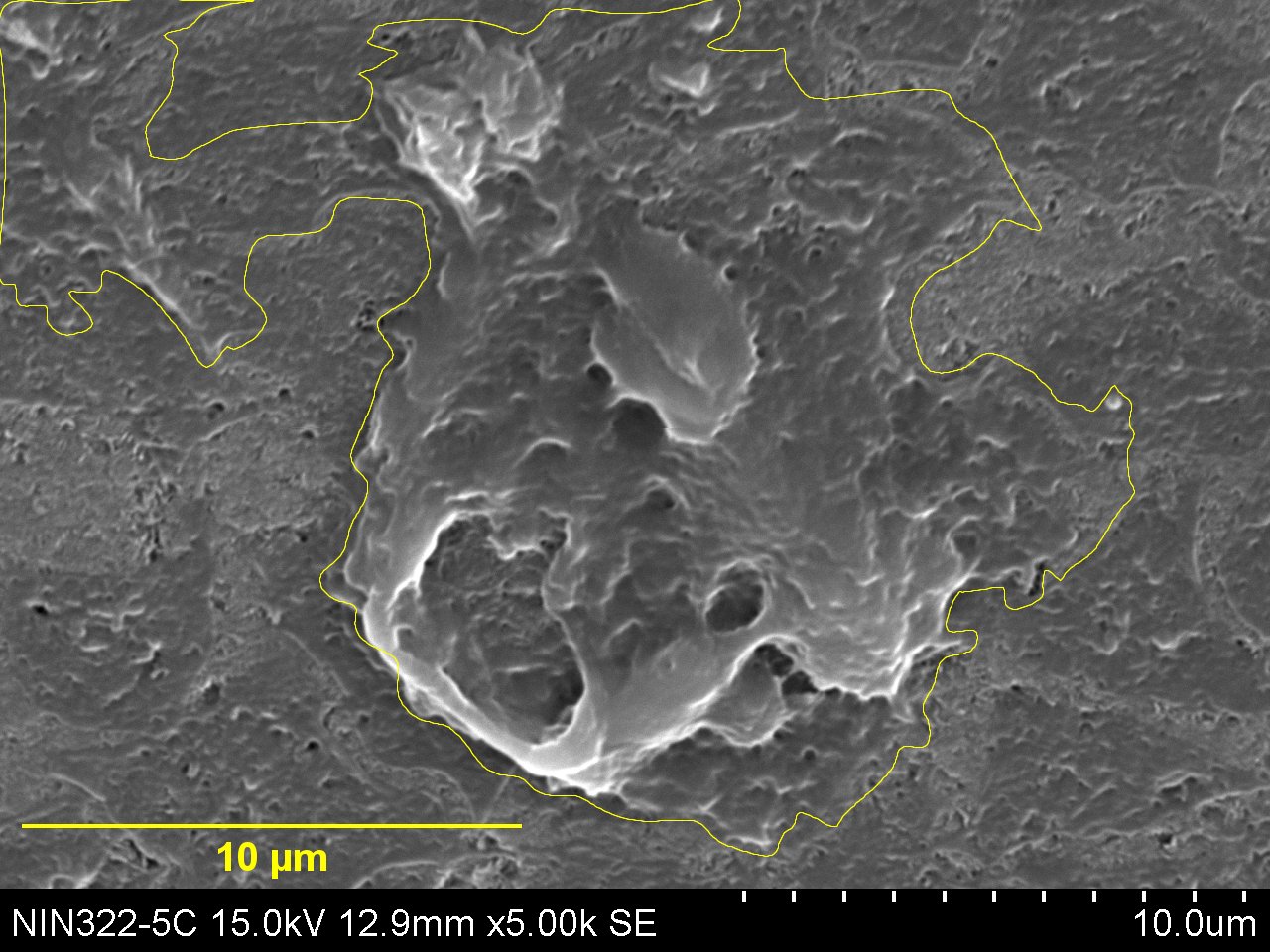

Supplement: S12 Fig — Amorphous deposits resembling RBC ghosts were seen on the valve. Total area = 452.173 μm2, platelet area = 0, RBC area = 0, Acellular deposit area = 219.492 μm2, Total area occupied by masses (cells and deposits)=219.492 μm2 (48.54%), Mass free area = 232.681 μm2 (51.46%), Magnification = 5000x, working distance = 12.9 mm. The scale bar is shown in the image for reference. (TIFF) [file pone.0336760.s012.tiff]

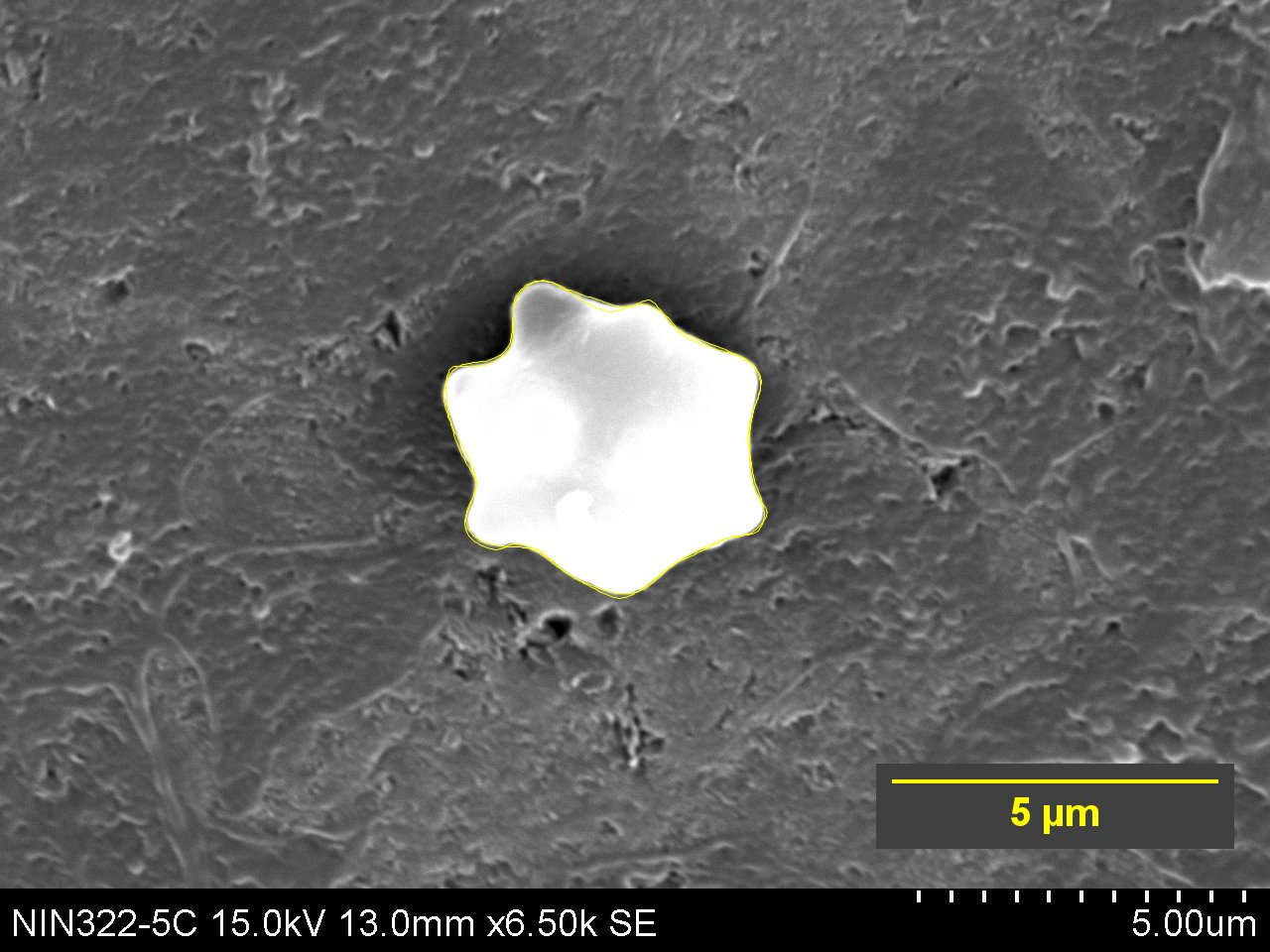

Supplement: S13 Fig — A single RBC with finger like cytoplasmic projections, presumably due to dehydration, is seen. Total area = 265.16 μm2, platelet area = 0, RBC area = 16.573 μm2, Acellular deposit area = 0, Total area occupied by masses (cells and deposits)=16.573 μm2 (6.25%), Mass free area = 248.587 μm2 (93.75%), Magnification = 6500x, working distance = 13 mm. The scale bar is shown in the image for reference. (TIFF) [file pone.0336760.s013.tiff]

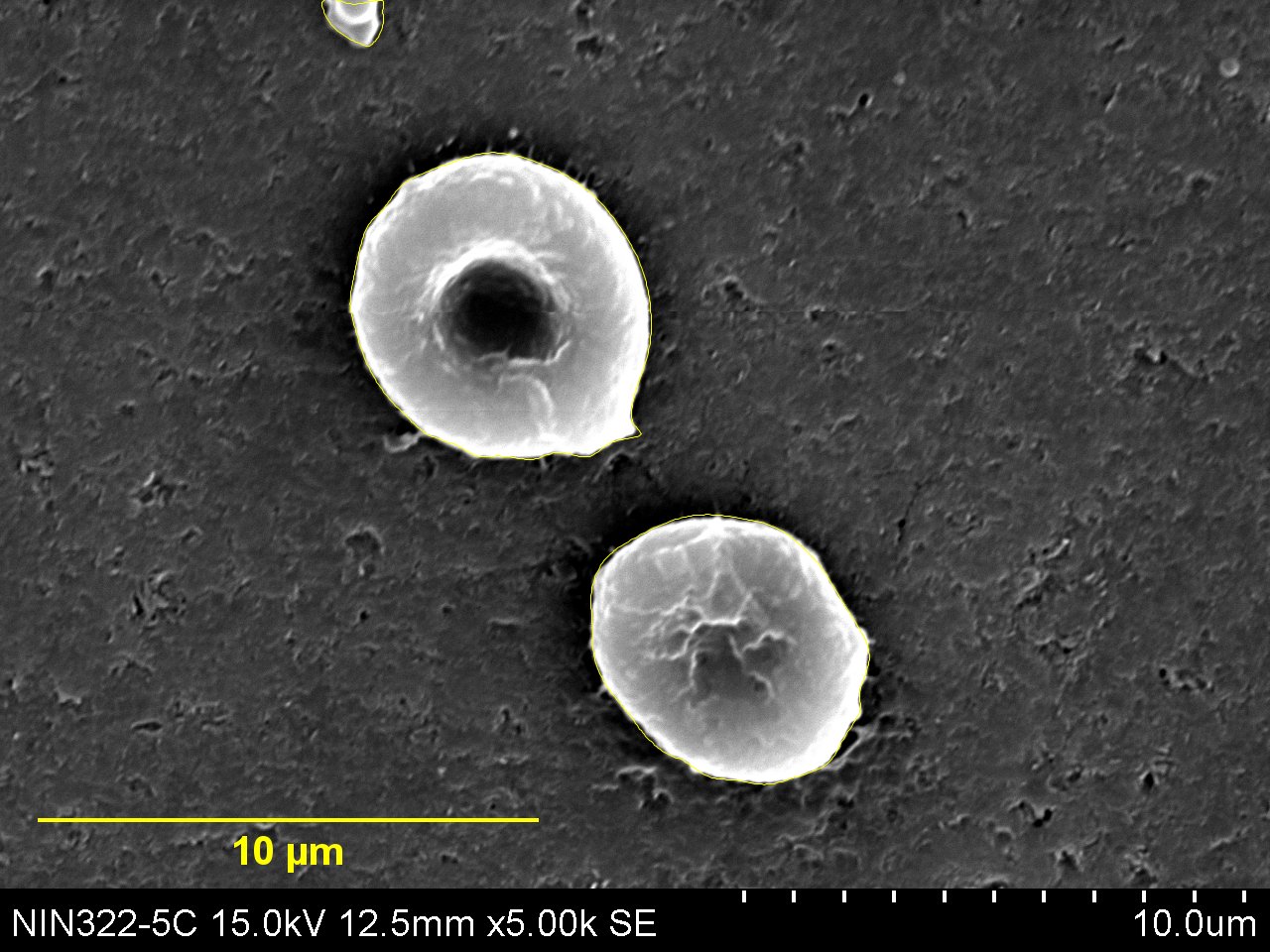

Supplement: S14 Fig — Two isolated RBCs with wrinkled membranes are seen. Total area = 450.215 μm2, platelet area = 0, RBC area = 51.721 μm2, Acellular deposit area = 0.807, Total area occupied by masses (cells and deposits)=52.528 μm2 (11.67%), Mass free area = 397.687 μm2 (88.33%), Magnification = 5000x, working distance = 13 mm. The scale bar is shown in the image for reference. (JPG) [file pone.0336760.s014.jpg]

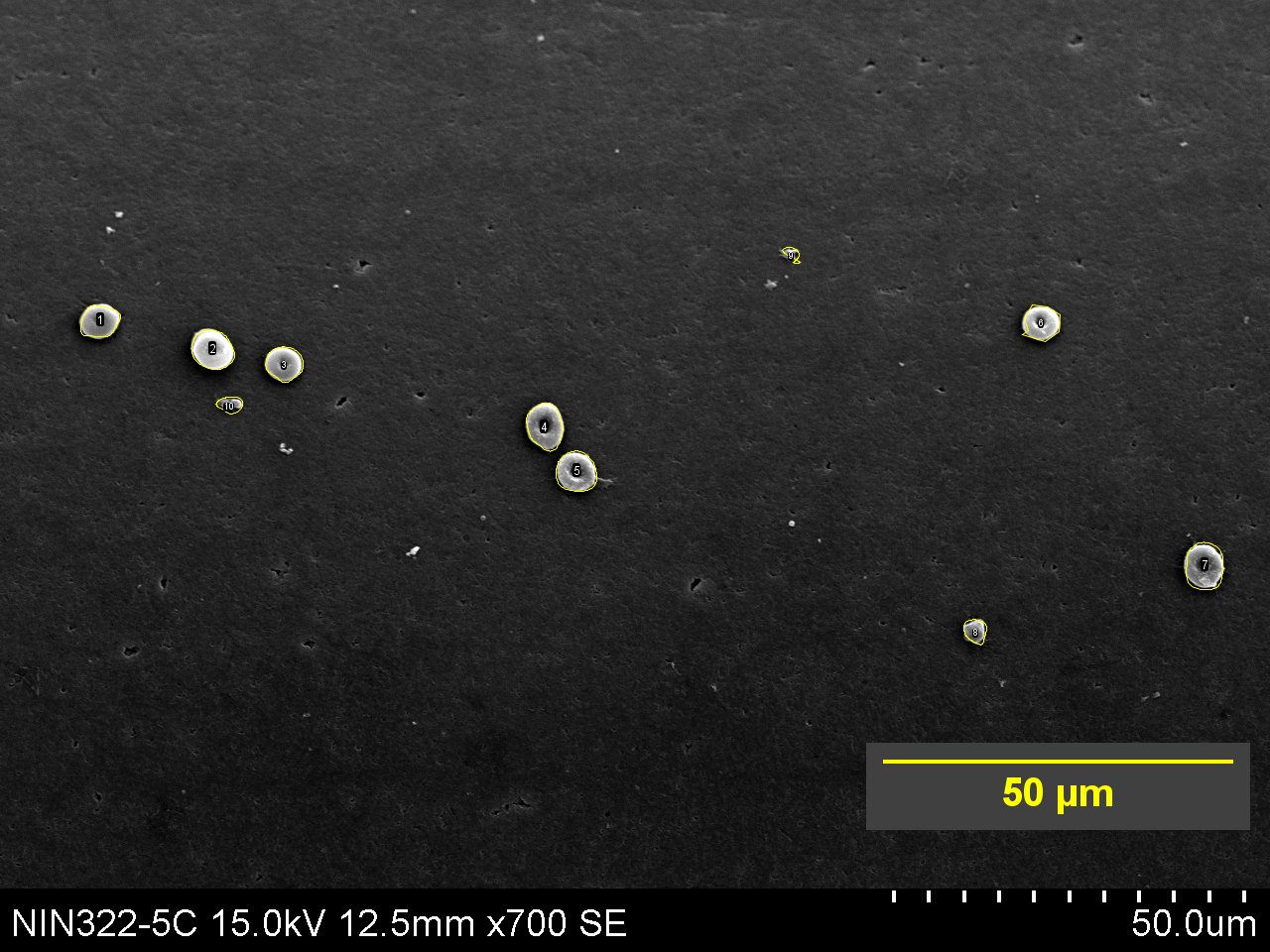

Supplement: S15 Fig — Few solitary RBCs and small deposits are seen, but much of the valve area is virtually deposit-free. Total area = 23034.515 μm2, platelet area = 0, RBC area = 169.504 μm2, Acellular deposit area = 18.334, Total area occupied by masses (cells and deposits)=187.838 μm2 (0.82%), Mass free area = 22846.68 μm2 (99.18%), Magnification = 700x, working distance = 12.5 mm. The scale bar is shown in the image for reference. (TIFF) [file pone.0336760.s015.tiff]

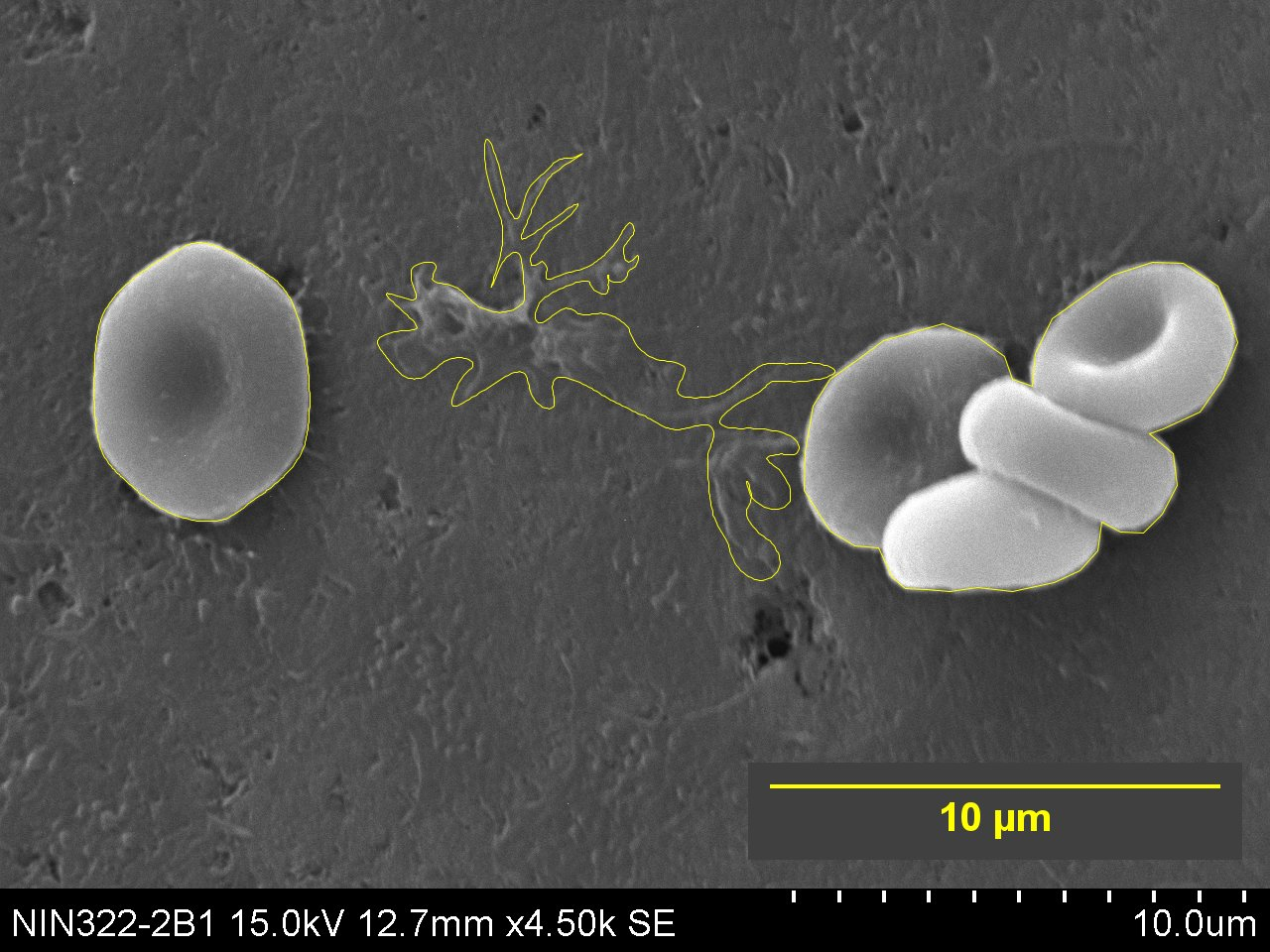

Supplement: S16 Fig — A solitary RBC and a cluster of RBCs are seen. One or more platelets in the spreading dendritic stage are seen. Total area = 561.36 μm2, platelet area = 20.105, RBC area = 68.779 μm2, Acellular deposit area = 0, Total area occupied by masses (cells and deposits)=88.88 μm2 (15.83%), Mass free area = 472.476 μm2 (84.17%), Magnification = 4500x, working distance = 12.7 mm. The scale bar is shown in the image for reference. (TIFF) [file pone.0336760.s016.tiff]

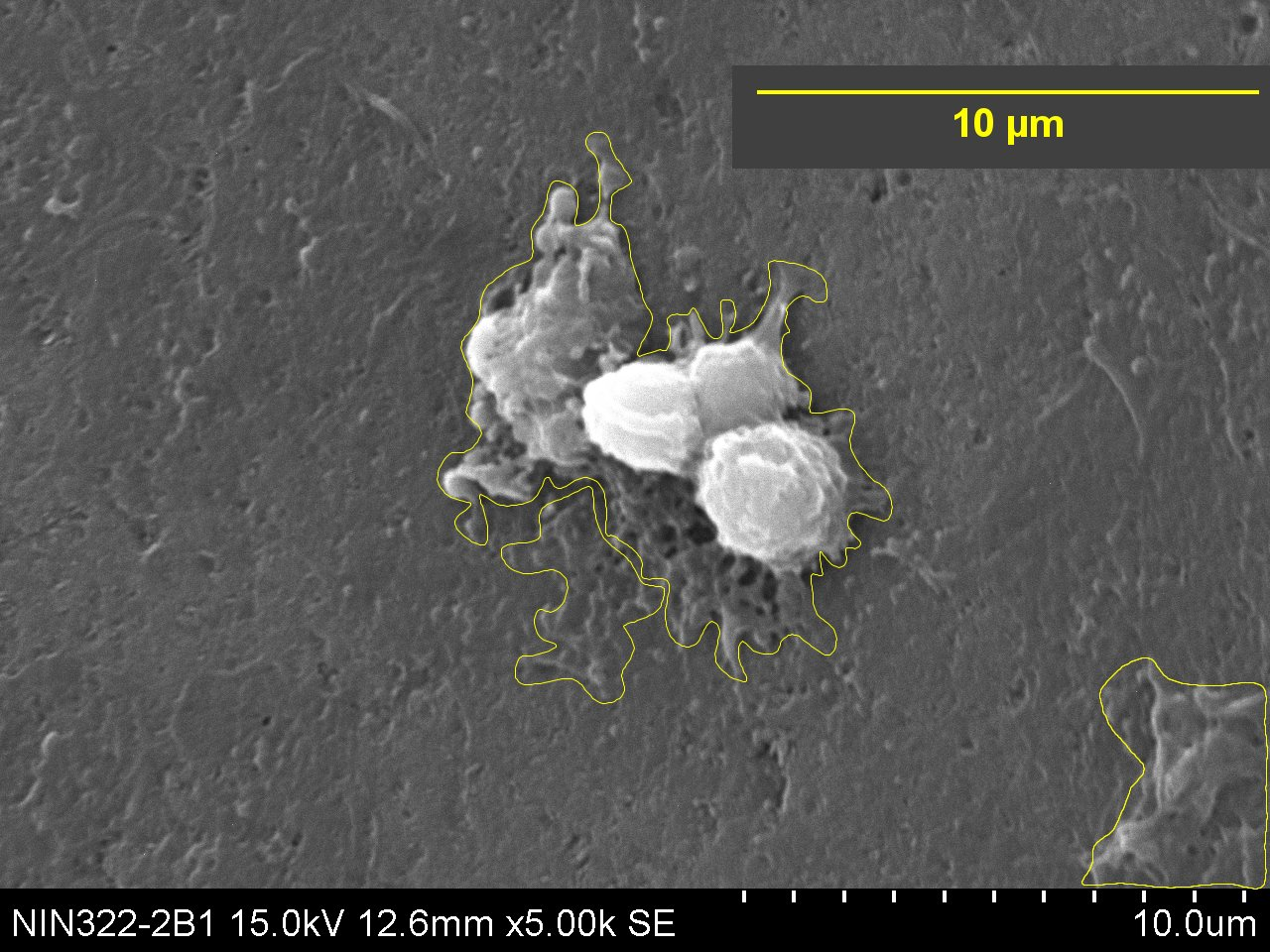

Supplement: S17 Fig — A cluster of activated platelets in the spreading dendritic stage is seen in the centre of the field. Total area = 447.435 μm2, platelet area = 44.362, RBC area = 0 μm2, Acellular deposit area = 19.548, Total area occupied by masses (cells and deposits)=63.91 μm2 (14.28%), Mass free area = 383.525 μm2 (85.72%), Magnification = 5000x, working distance = 12.6 mm. The scale bar is shown in the image for reference. (TIFF) [file pone.0336760.s017.tiff]

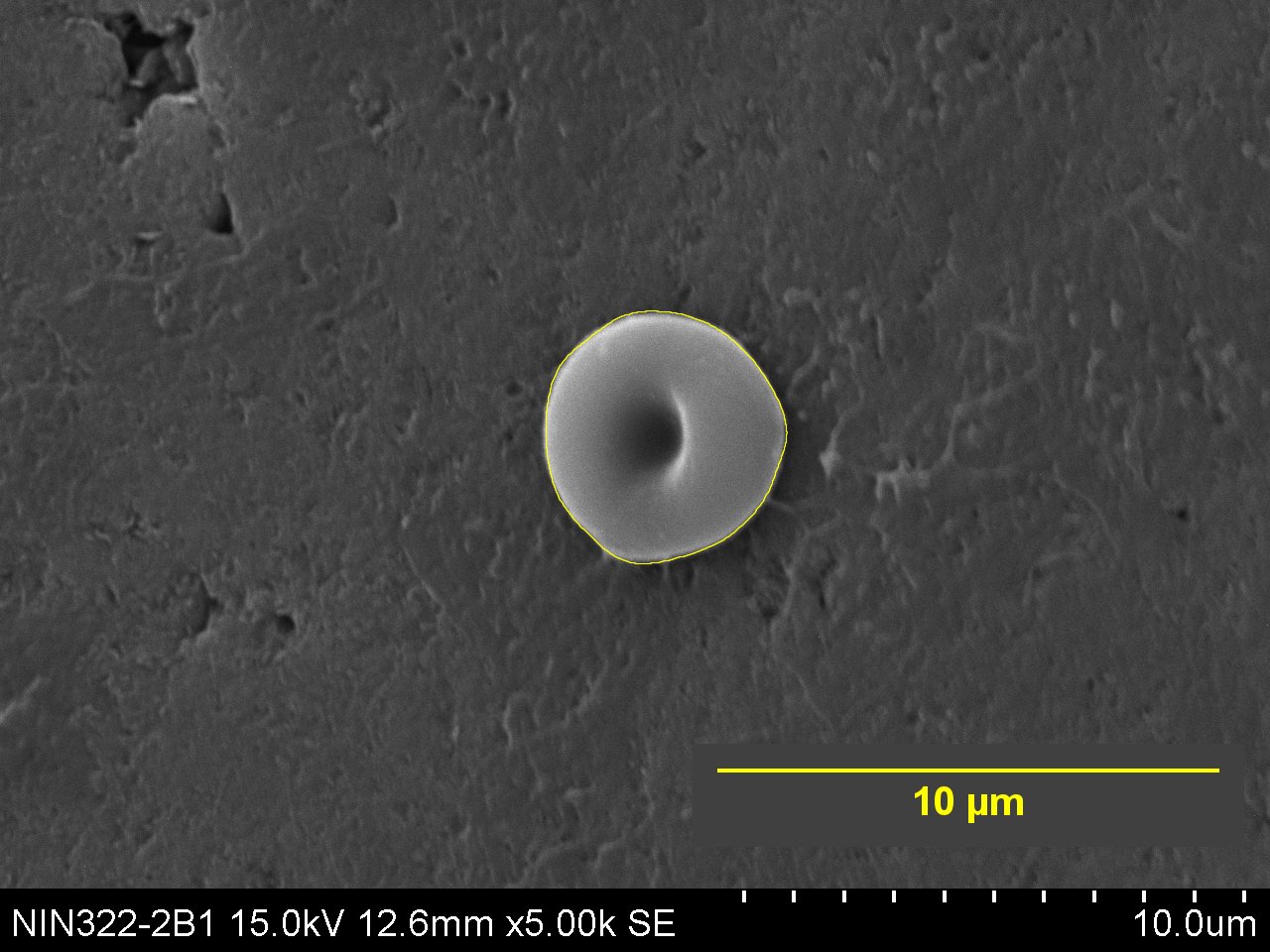

Supplement: S18 Fig — A solitary RBC is seen in the center of the field. Total area = 447.921 μm2, platelet area = 0, RBC area = 18.494 μm2, Acellular deposit area = 0, Total area occupied by masses (cells and deposits)=18.494 μm2 (4.13%), Mass free area = 429.427 μm2 (95.87%), Magnification = 5000x, working distance = 12.6 mm. The scale bar is shown in the image for reference. (TIFF) [file pone.0336760.s018.tiff]

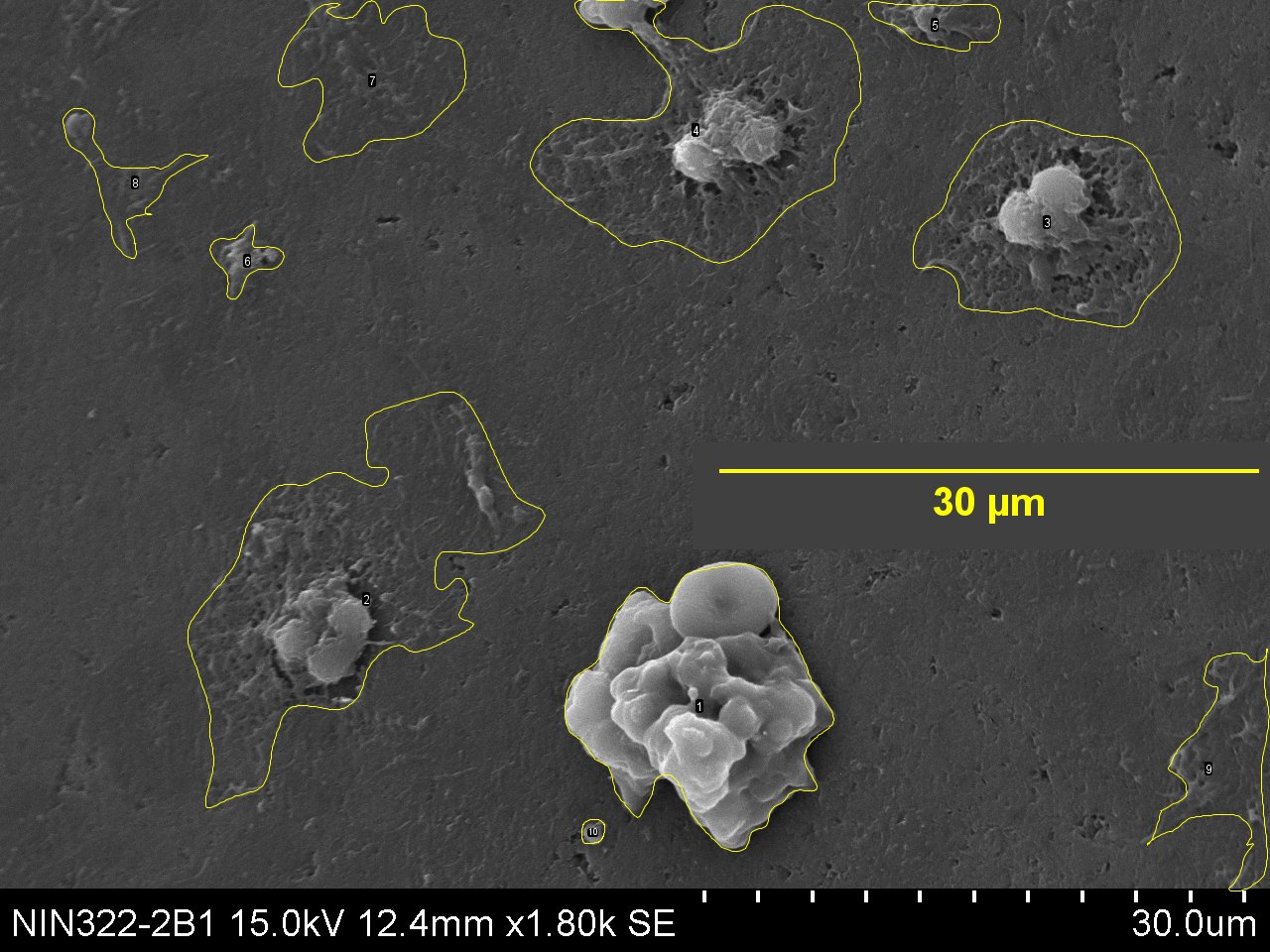

Supplement: S19 Fig — A large cluster of deformed RBCs is seen in the lower half of the field. At least three clusters of adherent and activated platelets in the spreading dendritic stage are seen in addition to focal amorphous deposits. Total area = 3491 μm2, platelet area = 508.738 μm2, RBC area = 149.973 μm2, Acellular deposit area = 85.18 μm2, Total area occupied by masses (cells and deposits)=743.891 μm2 (21.31%), Mass free area = 2747.109 μm2 (78.69%), Magnification = 1800x, working distance = 12.4 mm. The scale bar is shown in the image for reference. (TIFF) [file pone.0336760.s019.tiff]

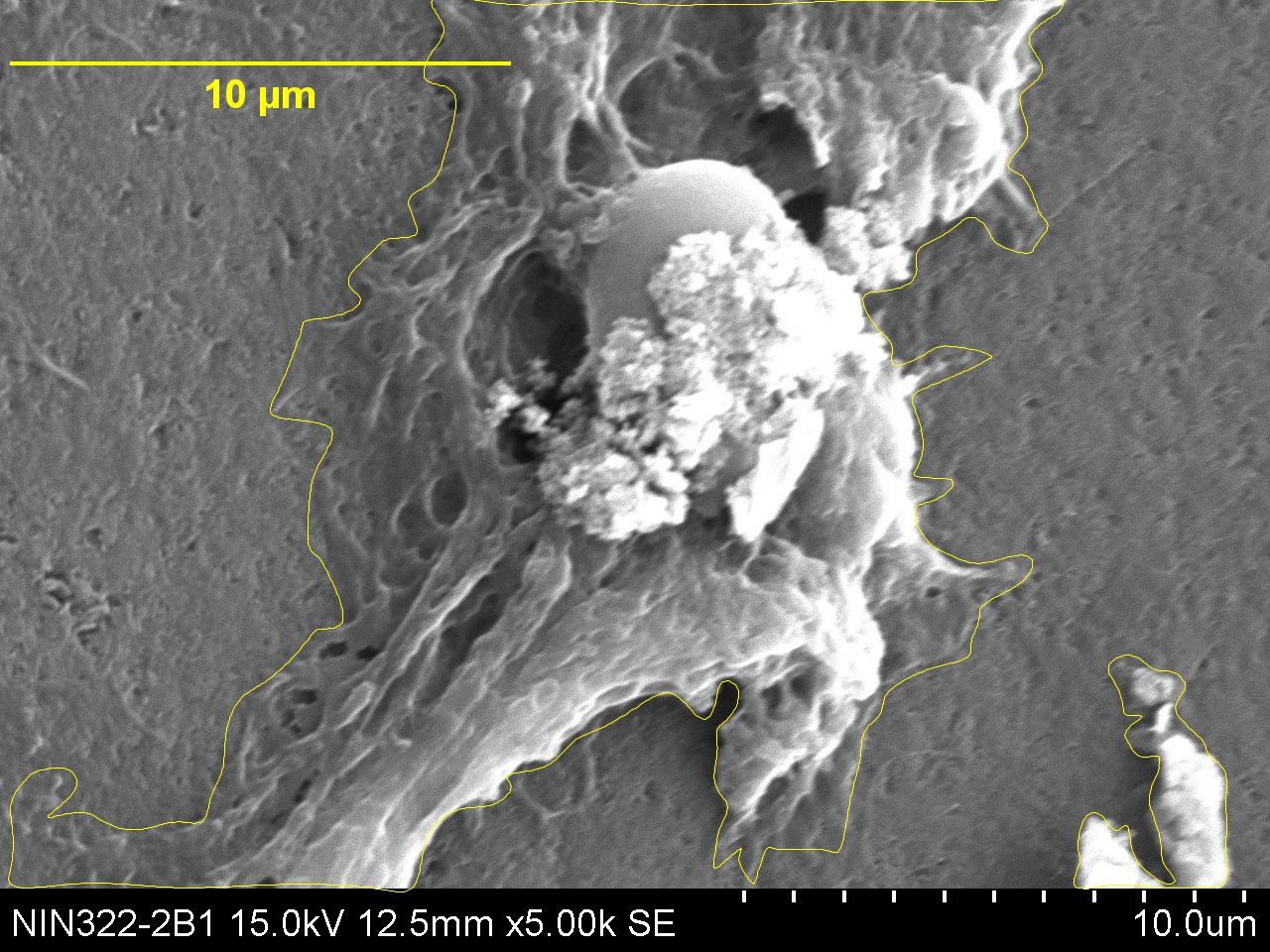

Supplement: S20 Fig — A larger activated platelet-rich deposit is seen in the field. Total area = 449.697 μm2, platelet area = 205.294 μm2, RBC area = 0, Acellular deposit area = 7.403 μm2, Total area occupied by masses (cells and deposits)=213.327 μm2 (47.44%), Mass free area = 236.37 μm2 (52.56%), Magnification = 5000x, working distance = 12.5 mm. The scale bar is shown in the image for reference. (TIFF) [file pone.0336760.s020.tiff]

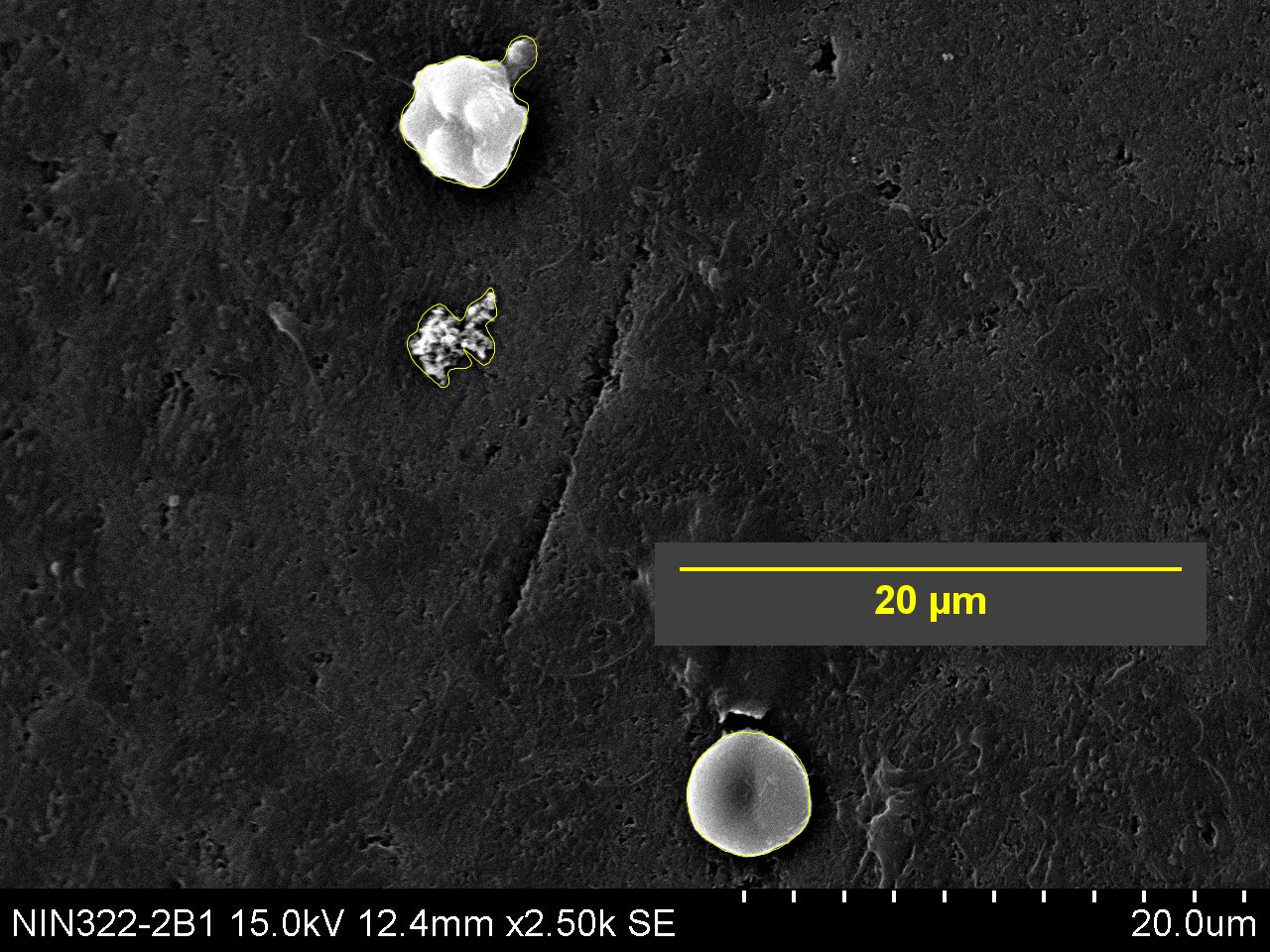

Supplement: S21 Fig — Two isolated swollen RBC are seen with focal amorphous deposits. Total area = 1793.741 μm2, platelet area = 0 μm2, RBC area = 39.984 μm2, Acellular deposit area = 7.977 μm2, Total area occupied by masses (cells and deposits)=47.961 μm2 (2.67%), Mass free area = 1745.78 μm2 (97.33%), Magnification = 2500, working distance = 12.4 mm. The scale bar is shown in the image for reference. (TIFF) [file pone.0336760.s021.tiff]

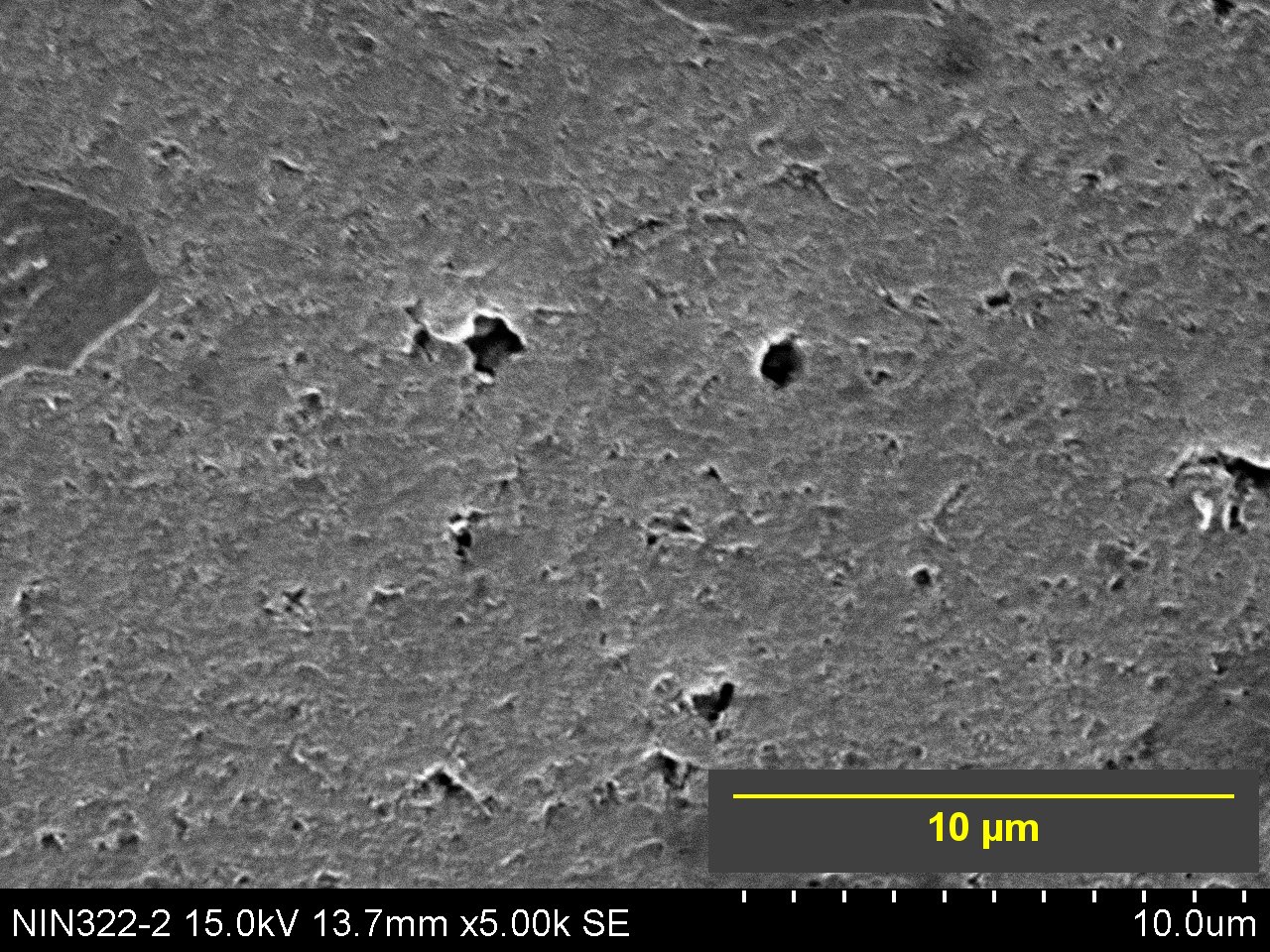

Supplement: S22 Fig — Total area = 448.409 μm2, platelet area = 0 μm2, RBC area = 0 μm2, Acellular deposit area = 0 μm2, Total area occupied by masses (cells and deposits)=0 (0%), Mass free area = 448.409 μm2 (100%), Magnification = 5000x, working distance = 13.7 mm. The scale bar is shown in the image for reference. (TIFF) [file pone.0336760.s022.tiff]

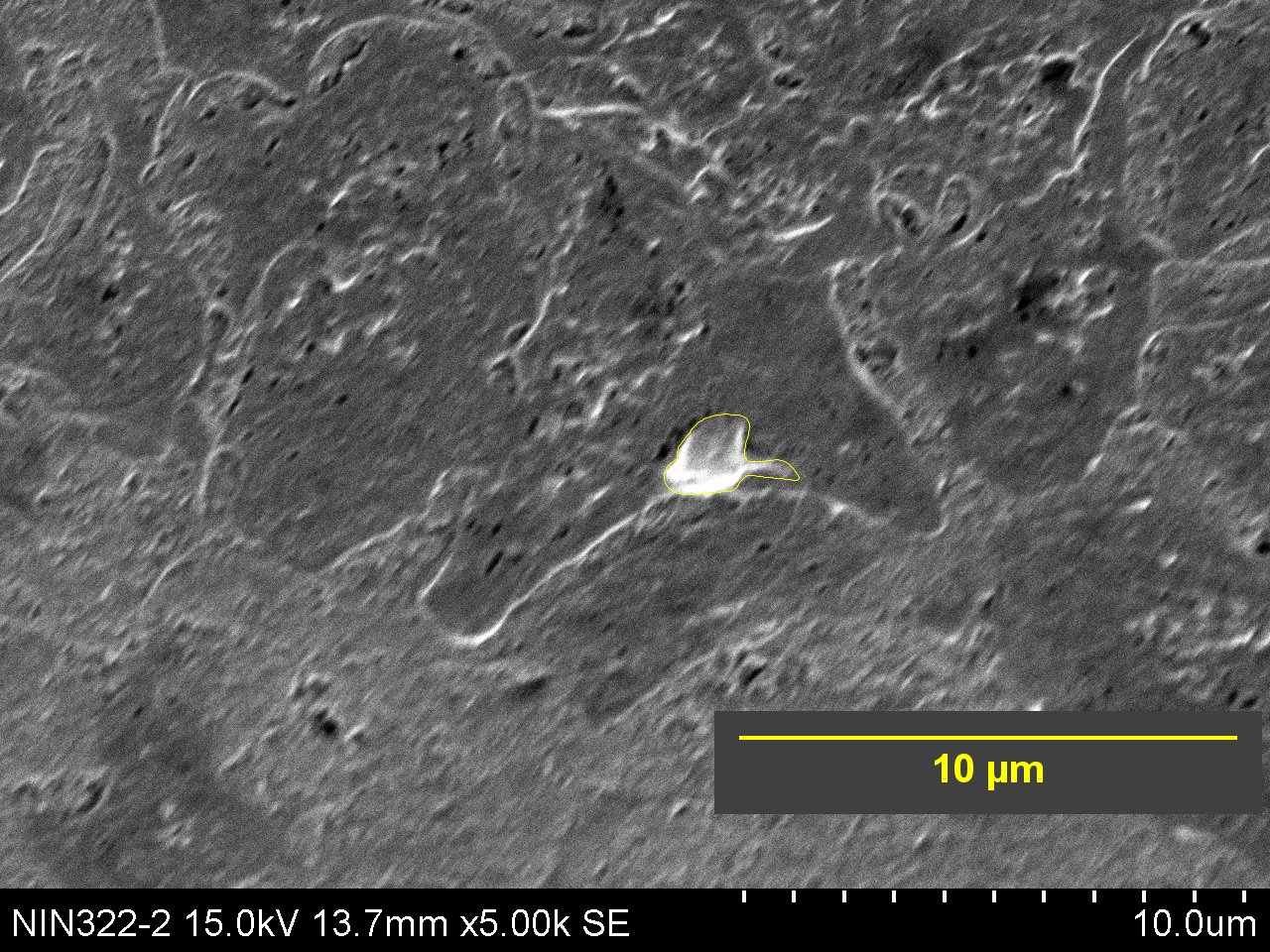

Supplement: S23 Fig — A single platelet extending its dendritic process, indicating the incipient stage of platelet activation. Total area = 454.589 μm2, platelet area = 2.379 μm2, RBC area = 0, Acellular deposit area = 0, Total area occupied by masses (cells and deposits)=2.379 μm2 (0.52%), Mass free area = 452.21 μm2 (99.48%), Magnification = 5000x, working distance = 13.7 mm. The scale bar is shown in the image for reference. (TIFF) [file pone.0336760.s023.tiff]

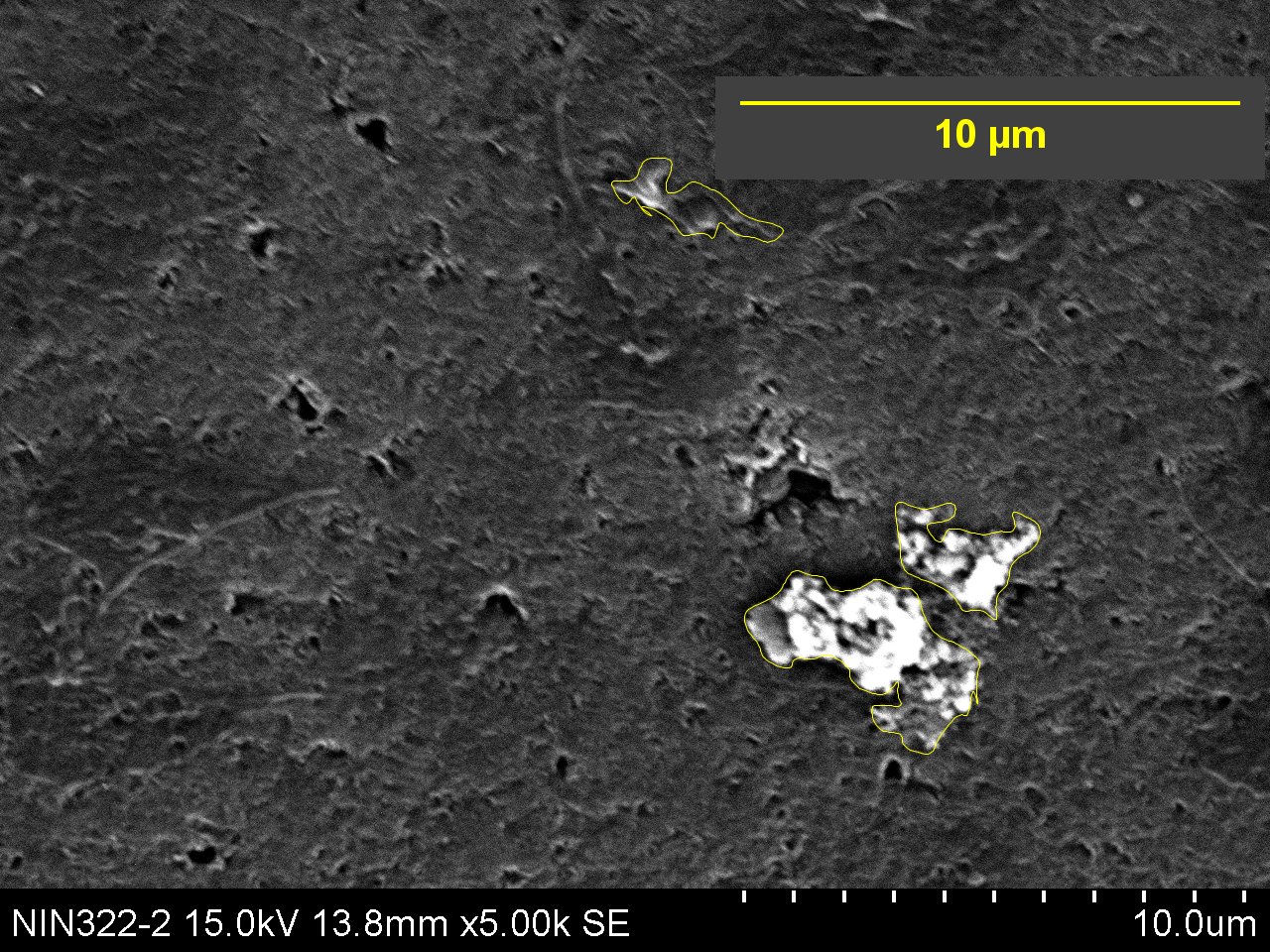

Supplement: S24 Fig — Focal amorphous deposits are seen. Total area = 451.483 μm2, platelet area = 0, RBC area = 0, Acellular deposit area = 14.404 μm2, Total area occupied by masses (cells and deposits)=14.404 μm2 (3.19%), Mass free area = 437.079 μm2 (96.81%), Magnification = 5000x, working distance = 13.8 mm. The scale bar is shown in the image for reference. (TIFF) [file pone.0336760.s024.tiff]

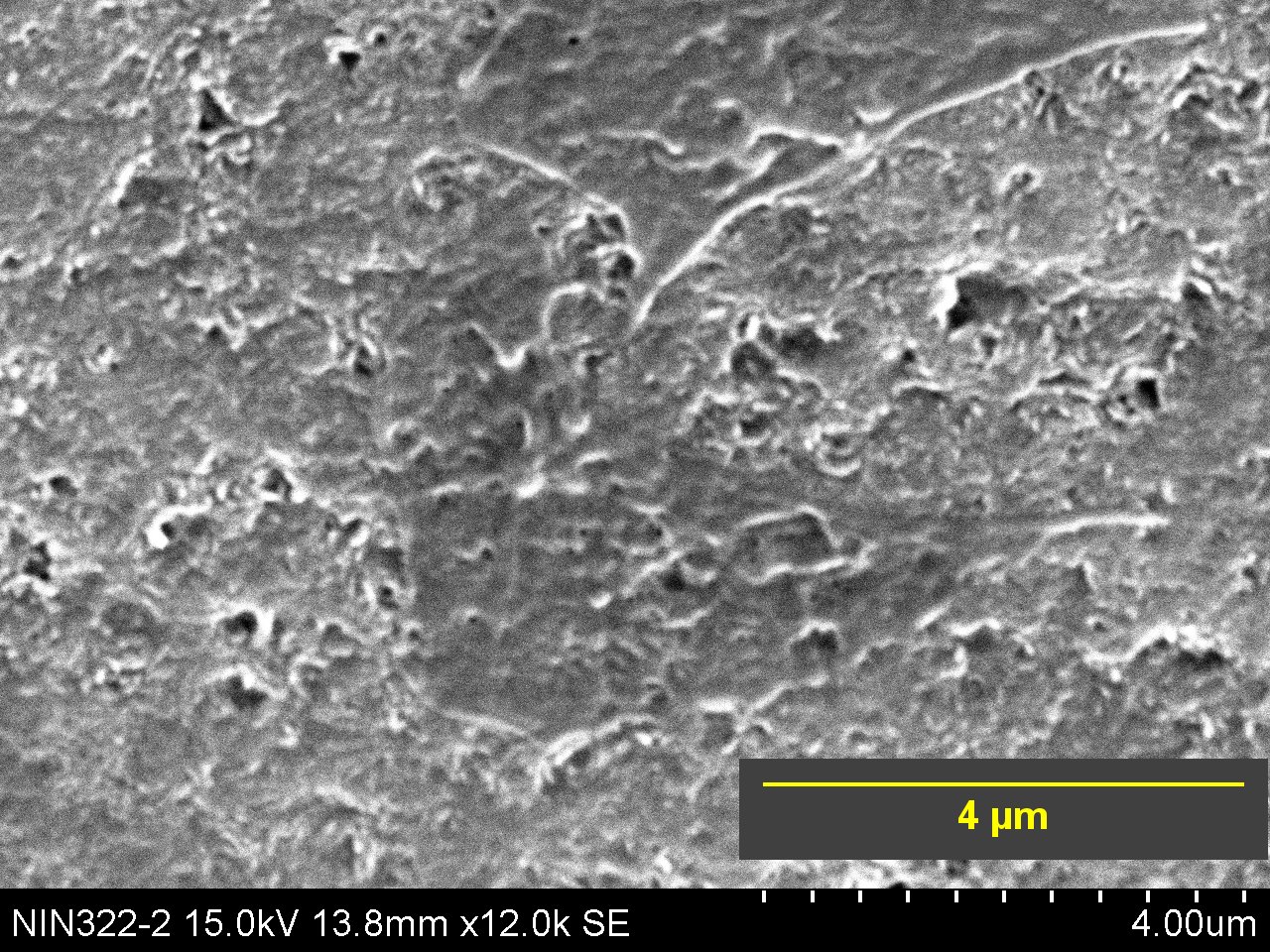

Supplement: S25 Fig — Total area = 77.924 μm2, platelet area = 0, RBC area = 0, Acellular deposit area = 14.404 μm2, Total area occupied by masses (cells and deposits)=0 (0%), Mass free area = 77.924 μm2 (100%), Magnification = 12000x, working distance = 13.8 mm. The scale bar is shown in the image for reference. (TIFF) [file pone.0336760.s025.tiff]
